# A Five-Year Review of Feto-Maternal Outcome of Antepartum Haemorrhage in a Tertiary Center

**DOI:** 10.23958/ijirms/vol08-i03/1637

**Published:** 2023-03-16

**Authors:** Charlotte B. Oguejiofor, Chidimma D. Okafor, George U. Eleje, Joseph I. Ikechebelu, Chigozie G. Okafor, Joseph O. Ugboaja, Chukwudi A. Ogabido, Tobechi K. Njoku, Osita S. Umeononihu, Boniface C. Okpala, Malarchy E. Nwankwo, Chijioke O. Ezeigwe, Chukwunonso I. Enechukwu, Ahizechukwu C. Eke

**Affiliations:** 1 Department of Obstetrics & Gynaecology, Nnamdi Azikiwe University Teaching Hospital Nnewi, PMB 5025, Nnewi, Anambra State, Nigeria.; 2 Effective Care Research Unit, Department of Obstetrics and Gynaecology, Nnamdi Azikiwe University, Nnewi Campus, Nigeria.; 3 Division of Maternal and Fetal Medicine, Department of Gynaecology and Obstetrics, Johns Hopkins University School of Medicine, Baltimore, USA.

**Keywords:** Fetal, Maternal, Outcome, Anterpartum-haemorrhage, Abruptio-placentae, Placenta-praevia

## Abstract

**Background:**

Pregnancies complicated with antepartum-haemorrhage is high risk pregnancies associated with adverse maternal, fetal-and-perinatal-outcomes. It contributes significantly to fetal and maternal mortality especially in the developing countries. Proper antenatal care and prompt intervention is necessary to forestall adverse and improve outcome.

**Objective:**

To determine the prevalence, sociodemographic characteristics, risk factors, fetomaternal outcome of pregnancies with antepartum haemorrhage.

**Methods:**

The case files of the patients were retrieved from the medical records department. The total number of deliveries within the study period was obtained from the labour ward records. The feto-maternal-outcome-measures were; prevalence of caesarean-section, postpartum-haemorrhage, hysterectomy, need for blood-transfusion, maternal-death, prematurity, need for admission in intensive-care-unit and still births. The data was analysed using SPSS version 21. Chi-square was used to test for significance.

**Results:**

Within the 5-year period under review, out of a total of 6974 deliveries, 234 had antepartum-haemorrhage (3.4% prevalence rate). Abruptio-placentae was the commonest cause and accounted for 69.5% of the cases (prevalence of 2.1%) while placenta praevia accounted for 28.2% of the cases (prevalence rate of 0.9%). The mean age of the women was 31.8±5.3 years. The mean parity was 3.4±1.7 and majority (63.8%) of the women were unbooked. The commonest identifiable risk factors were multiparity and advanced maternal age. One-hundred-and sixty-six (77.9%) women were delivered through the abdominal route. Postpartum-haemorrhage occurred in 22.1% (47) of the cases while prematurity was the commonest fetal complications. Maternal mortality was 0.47% (1) while still birth was 44.1% (94).

**Conclusion:**

There is high prevalence of antepartum-haemorrhage in our environment. Abruptio-placentae was the commonest cause and associated with significant adverse fetomaternal-outcome when compared with placenta-praevia. Thus, good and quality antenatal care as well as high index of suspicion, prompt diagnosis and treatment remain the key to forestall these complications and improve fetomaternal-outcome.

## Introduction

Antepartum haemorrhage (APH) is a major cause of fetomaternal morbidity and mortality ^[1]^. It is defined as bleeding from the genital tract during pregnancy, after the age of viability until the delivery of the fetus (end of second stage) ^[2]^. Age of viability is after 20 weeks of gestation in industrialized countries and 28 weeks in low resource countries lacking adequate neonatal facilities ^[3]^. It is an obstetric emergency with a prevalence of 0.5-5% ^[1]^.

The main causes of ante-partum haemorrhage are placenta praevia and abruptio placenta. The other causes include heavy show, vasa praevia, cervicitis, genital trauma, varicosities, tumours, infections, coagulation defects and may be unknown in some cases ^[3]^. The risk factors for APH include chronic maternal hypertension, pre-eclampsia, multiparity, maternal smoking, history of previous caesarean birth and increased maternal age ^[4]^.

APH is associated with increased adverse maternal complications ranging from premature labour, postpartum hemorrhage, shock, sepsis, retained placenta, increased rate of cesarean section, peripartum hysterectomies, coagulation failure and even death ^[5]^. Fetal complications are premature delivery, low birth weight, intrauterine fetal death and birth asphyxia ^[5,6]^. APH contributes significantly to maternal and neonatal morbidity and mortality in sub-Saharan Africa.

APH has not been studied in its entirety in our center, the closest studies were on abruptio placentae alone by Eleje *et al*^[7]^ and placenta praevia by Ikechebelu *et al*^[8]^ done about 5 and 10 years ago respectively, thus necessitating the need for this study and also to update the record on abruptio placentae and placenta praevia. The study was aimed at assessing the fetomaternal outcome of pregnant women presenting with APH in NAUTH, Nnewi.

## Materials and Method

### Study Design:

This is a 5-year retrospective study of pregnant women that were managed for antepartum haemorrhage.

### Study Setting:

This study was conducted at the Nnamdi Azikiwe University Teaching Hospital (NAUTH), Nnewi, Nigeria between 1st April 2012 and 31st March 2017. NAUTH is a 400 bedded tertiary center located in South Eastern part of Nigeria. It provides excellent out-patient obstetric services as well as obstetric emergency services on a 24hour basis, specialized and comprehensive health services to the inhabitants of Anambra state and parts of the surrounding States.

Provides out-patient obstetric services on every working day of the week as well as obstetric emergency services on a 24hour basis.

The labour ward admission register was assessed to determine the total number of deliveries within the study period. The folder numbers of the patients with antepartum haemorrhage were obtained from the delivery register, clinic registers and theatre register. The case files were retrieved from Medical Records Department of the hospital. Out of the 6974 deliveries, 234 cases of antepartum haemorrhage were managed. A total of 221 folders were retrieved giving a retrieval rate of 94.4%. Only 213 had the necessary information needed for the study and thus were used for this study. The patients were analyzed for variables such as age, parity, marital status, booking status, gestational age at delivery, recognized risk factors, route of delivery, perinatal and maternal outcome. The data was analyzed using SPSS version 21. Chi-square was used to test for significance. P<0.05 was taken to be significant.

## Results

Within the 5-year period under review, a total of 6,974 deliveries were conducted in our hospital. The total number of cases of antepartum haemorrhage delivered was 234 giving a prevalence of 3.4% or 34 per 1000 deliveries for antepartum haemorrhage. Abruptio placentae is the commonest cause of antepartum haemoarrhage in the study and accounted for 69.5% of the cases with the prevalence of 2.1% while placenta praevia accounted for 28.2% of the cases with the prevalence rate of 0.9% (Table 3). Only 213 case files were used for analysis. The ages of the women ranged from 22 to 43years, the mean age of the women was 31.8± 5.3 years with the 30-34years age group being the commonest 72(33.8%) (Table 1). All the patients were married. Seventy-seven (36.2%) of them were booked while 136(63.8%) were unbooked (Table 1). The parity of the women ranged from 0 to 8 with mean parity of 3.4±1.7 (Table 1). The gestational age ranged from 28 to 43 weeks (Table 1). Sixty-six (31.0%) of the women presented at term while 147 (69%) were preterm. The identified risk factors are as shown in table 1. The commonest risk factors identified were advanced maternal age and high parity. Seventy-two (33.8%) of the women presented with anaemia (Table 1). Caesarean section was the commonest route of delivery and emergency caesarean section accounted for 51.2% of the cases. A total of 12 (5.6%) patients had hysterectomy (Table 2). The commonest fetal/neonatal complication was prematurity while 69 (32.4%) of the babies delivered were admitted into neonatal intensive care unit. Maternal mortality was 1(0.47%) while perinatal mortality was 94(44.1%) (Table 2).

Comparison of fetomaternal outcomes in the patients with abruptio placentae were summarised in table 4. It showed that number of stillbirths and babies delivered preterm were significantly higher in patients with abruptio placentae than in placenta praevia (*χ*^2^= 44.051, p<0.001 and *χ*^2^=30.180, p<0.001 respectively). The number of babies with low birth weight and those delivered via caesarean section were significantly higher in patients with abruptio placentae than in placenta praevia (*χ*^2^=13.335, p<0.001 and *χ*^2^=144.21, p<0.001). The number of babies that required NICU admission were 41 (62.1%) for abruptio placentae and 25 (37.9%) for placenta praevia. Abruptio placentae accounted for 78.3% (36) of the patients that had primary postpartum haemorrhage. All the patients that had hysterectomy were patients with abruptio placentae. Female babies were commoner in patients with abruptio placentae than in placenta praevia.

## Discussion

The prevalence of antepartum haemorrhage in this study was 3.4%. Abruptio placentae was the commonest cause and accounted for 69.5% of the cases with the prevalence of 2.1% while placenta praevia accounted for 28.2% of the cases with the prevalence rate of 0.9%. This is comparable to antepartum haemorrhage prevalence of 3.5% and 3.8% reported in Lagos and India respectively ^[2,9,10]^. A lower prevalence of 1.2% was reported in Kano and higher prevalence rates of 5.1% and 15.3% reported in Ethiopia and Qatar respectively ^[4,11]^. The commonest cause of APH in Kano, Ethiopia and India was abruptio placentae while placenta praevia was the commonest cause in Lagos and Greece ^[3,10-13]^. The prevalence rate of abruptio placentae in Tanzania was 2.5% and similar to the rate got in this study ^[14]^. The prevalence of placenta praevia in Maiduguri was 0.8% and thus similar to the prevalence rate of 0.9% in this study ^[15]^. However, the prevalence of abruptio placentae reported in Nnewi was 0.8% 5 years ago ^[7]^. The high prevalence of abruptio placentae in the study could be due to anecdotal report of increase in the hypertensive disorders in the institution, better record keeping and the fact that the institution serves a referral center with the capacity to salvage preterm babies, thus facilitating referral from neighbouring communities.

Maternal age of 35 years and above and high parity were the commonest risk factors identified with each accounting for 28.6% (61) of cases of APH studied. This is similar to the finding in Kano ^[7]^. Another study in Pakistan found that most of the women studied were aged 35 and above and majority (62%) of them were grandmultipara ^[16]^. Other risk factors identified in the study were hypertensive disorders in pregnancy (16%), previous uterine instrumentation (16%), previous abortion (23.5%), previous caesaerean section (11.7%) and multiple gestation (5.2%). Similar findings were reported by other studies ^[17]^.

The prevalence of APH in multiparous women was 71.3% when compared with 28.7% for low parity women. Grandmultiparity alone accounted for 28.6% of the cases. Similar findings were reported in Lagos, Kano and Tanzania ^[3,11,16]^. This shows that APH is predominantly a disease of the multipara. Therefore, right application of family planning and contraception will reduce the prevalence of this condition and thus forestall the complications associated with it.

Majority of the patients with APH in this study were unbooked and accounted for 63.8% (136) of the cases. The prevalence of APH in unbooked women in Kano was 63.3% and thus similar to the finding in our center. This obviously underscores the need for quality antenatal care as it helps in identifying the risk factors, early detection or diagnosis and prompt treatment. The role of antenatal in preventing the complications associated with antepartum haemorrhage cannot be overemphasized as it gives opportunity for health education, counseling and risk factors identification. Even though bleeding can cause anaemia, poor antenatal care may have contributed to the reason one-third (33.8%) of the women with APH presented with anaemia in the study. Sharmila et al reported similar finding ^[10]^. Where antenatal care is lacking, pregnancy becomes at risk of complications including APH and its sequalae as demonstrated in this study.

Pregnancy outcome in APH is associated with fetal and maternal complications. It has been implicated as a major cause of fetomaternal morbidity and mortality ^[18]^. This study shows that women with APH are likely to have adverse fetomaternal outcome. The fetal outcome observed in this study include low birth weight, prematurity, increased rate of NICU admission and stillbirth which were 61.5%, 69%, 32.4% and 44.1% respectively. Majority (68.7%) of the women in this study delivered at the age between 28weeks and 32weeks. In India the commonest reported gestational age at delivery was between 31 weeks and 34 weeks ^[10]^. The commonest fetal outcome were prematurity and low birth weight accounting for 69% (147) and 61% (131) of cases of APH respectively. Abruptio placentae was responsible for 82.4% and 80.3% of prematurity and low birth weight respectively. Similar finding was reported in Tanzania ^[14]^. A higher prematurity rate of 82.8% was reported in India ^[10]^. Sixty-nine (32.4%) babies required NICU admission and abruptio placentae accounted 62.1% while still birth was the outcome of 44.4% (94) of the deliveries of the women diagnosed with APH. Prematurity and perinatal mortality was reported as the commonest fetal complications in Maiduguri ^[15]^. Lower prematurity rate of 7.7% was reported in Greece ^[13]^. Similar rate of stillbirth found in this study was reported in Kano and Tanzania ^[11,14]^. Lower still birth rates of 19.2%, 12.6% and 30.6% were reported in Lagos, India and Ethiopia ^[9,12,17]^. NICU admission was lower in the work done by Sharmila et al and accounted for 8.5% of the cases ^[10]^.

Abruptio placentae accounted for 94.6% of the still birth encountered in this study. This may be as a result of late presentation to the hospital at the onset of symptoms, thus predisposing the fetus to hypoxia and eventual death. This further buttress the need for health education on symptoms and early presentation to the hospital. Antenatal care provides an opportunity for such health education and counselling.

Majority of the deliveries were via caesarean section, accounting for 77.9% with abruptio placentae responsible in 87.3% of the cases. Studies done in Lagos and Kano and Maiduguri collaborated the finding that Caesarean section is the commonest route of delivery in APH though the reported rates were slightly lower than the rate found in this study ^[3,11,15]^. In contrast, placenta praevia was reported in India to account for 89.5 to 92.85% of caesarean sections done for APH ^[10,17]^. The prevalence of caesarean section in patients with abruptio placentae when compared with placenta praevia in this study could be as a result of pattern of referral as the majority of the cases studied were abruptio placentae. Abruptio placentae especially the severe ones are an obstetric emergency moreso when the baby is alive. Delivery is usually through the fastest and safest route and most of the times emergency caesaerean section becomes the only available option to prevent fetomaternal complications.

Forty-seven (22.7%) of the studied population had primary postpartum haemorrhage, 12 (5.2%) had hypovolaemic shock, 31.5% required blood transfusion and 5.6% of the patients had hysterectomy. Abruptio placentae was responsible for 80.6% of the transfusion and 100% of the hysterectomies. A study in Kano found similar rate of primary postpartum haemorrhage (24.2%) but a different rate of blood transfusion (61.5%) ^[11]^. Similar blood transfusion rate of 31.39% was reported India ^[17]^. The observed postpartum haemorrhage in this study could as a result of prevalent abruptio placentae cases and the fact that majority of the women are of high parity thus predisposing them to primary postpartum haemorrhage. One maternal death from complications of abruptio placentae was recorded within the period of this study. Maternal mortality was higher in Lagos and a rate of 3.1% was reported in Ethiopia ^[9,12]^.

## Conclusion

In conclusion, APH prevalence is high in our environment when compared with the findings from other institutions within the country. Abruptio placentae has been shown to be more common and associated with significant adverse fetomaternal outcome when compared with placenta praevia. Thus, good and quality antenatal care as well as high index of suspicion, prompt diagnosis and treatment remain the key to forestall these complications and improve fetomaternal outcome.

## Figures and Tables

**Table 1:** Socio-demographic characteristics

		Frequency	Percentage (%)
**Age**			
	20 – 24	15	7.0
	25 – 29	65	30.5
	30 – 34	72	33.8
	≥35	61	28.6
**Booking status**			
	Booked	77	36.2
	Unbooked	136	63.8
**Parity**			
	0 (Nullipara)	40	18.8
	1-4	112	52.6
	≥5	61	28.6
**Gestational Age**			
	28 – 32	101	47.4
	33 – 36	46	21.6
	≥37	66	31.0
**Risk factor**			
	Age (≥35 years)	61	28.6
	High parity (grandmultiparity)	61	28.6
	Hypertensive disorders in pregnancy	34	16
	Previous caesaerean section	25	11.7
	Previous uterine instrumentation	34	16
	Previous history of abortion	50	23.5
	Multiple pregnancy	11	5.2
**PCV (at presentation)**			
	Anaemic (<30)	72	33.8
	Normal (≥30)	141	66.2

PCV = Packed cell volume

**Table 2:** Feto-maternal Outcome

Maternal Outcome		Frequency	Percentage
Hysterectomy		12	5.6
Route of delivery	Elective C/S	45	21.1
	Emergency C/S	121	56.8
	SVD	47	22.1
PPH		47	22.1
Blood transfusion		67	31.5
Hypovolaemic shock		12	5.6
Maternal death		1	0.47
**Fetal Outcome**			
Low birth weight		131	61.5
Stillbirth		94	44.1
Prematurity		147	69
NICU admission		69	32.4
Sex distribution Male		80	37.6
Female		133	62.4

SVD = Spontaneous vertex delivery, NICU = Neonatal intensive care unit, C/S = Caesarean section, PPH = Postpartum haemorrhag

**Table 3:** Causes of antepartum haemorrhage

Type of APH		Frequency	Percent
	**Abruptio placentae**	148	69.5
	**Placenta praevia**	60	28.2
	**Others**	5	2.3
	**Total**	213	100.0

APH = Antepartum haemorrhage

**Table 4:** Comparison of fetomaternal outcome between patients with abruptio placentae and placenta praevia

Outcome	Abruptio placentae (%)	Placenta previa (%)	*χ*^2^ and *P*
**Birth outcome**			
Live birth	61 (52.6)	55 (47.4)	44.051,
Stillbirth	87 (94.6)	5 (5.4)	<0.001
**Sex**			
Male	46 (60.5)	30 (39.5)	6.590,
Female	102 (77.3)	30 (22.7)	0.010
**Birth weight**			
Low birth weight	102 (80.3)	25 (19.7)	13.335,
Normal	46 (56.8)	35 (43.2)	<0.001
**GA at delivery** (weeks)			
28-32	75 (78.1)	21 (21.9)	30.180,
33-36	42 (91.3)	4 (8.7)	<0.001
≥37	31 (47)	35 (53)	
**NICU Admission**			
Admitted	41 (62.1)	25 (37.9)	3.843,
None	107 (75.4)	35 (24.6)	0.050
**Mode of delivery**			
Elective C/S	0	45 (100.0)	144.21,
Emergency C/S	103 (87.3)	15(12.7)	<0.001
Vaginal	45 (100)	0	
**Blood transfusion**			
Transfusion	54 (80.6)	13 (19.4)	4.294,
None	94 (66.7)	47 (33.3)	0.038
**PPH**			
Present	36 (78.3)	10 (21.7)	
None	112 (69.1)	50 (30.9)	
**Hysterectomy**			
Present	11 (100.0)	0	4.708,
None	137 (69.5)	60 (30.5)	0.030
**Death**	1	0	

GA = Gestational age, NICU = Neonatal intensive care unit, C/S = Caesarean section, PPH = Postpartum haemorrhage
